# TRIMming down Flavivirus Infections

**DOI:** 10.3390/v16081262

**Published:** 2024-08-06

**Authors:** Marion Cannac, Sébastien Nisole

**Affiliations:** Institut de Recherche en Infectiologie de Montpellier (IRIM), Université de Montpellier, CNRS, 34090 Montpellier, France

**Keywords:** antiviral innate immunity, TRIM proteins, interferon response, flaviviruses, restriction factors

## Abstract

Flaviviruses comprise a large number of arthropod-borne viruses, some of which are associated with life-threatening diseases. Flavivirus infections are rising worldwide, mainly due to the proliferation and geographical expansion of their vectors. The main human pathogens are mosquito-borne flaviviruses, including dengue virus, Zika virus, and West Nile virus, but tick-borne flaviviruses are also emerging. As with any viral infection, the body’s first line of defense against flavivirus infections is the innate immune defense, of which type I interferon is the armed wing. This cytokine exerts its antiviral activity by triggering the synthesis of hundreds of interferon-induced genes (ISGs), whose products can prevent infection. Among the ISGs that inhibit flavivirus replication, certain tripartite motif (TRIM) proteins have been identified. Although involved in other biological processes, TRIMs constitute a large family of antiviral proteins active on a wide range of viruses. Furthermore, whereas some TRIM proteins directly block viral replication, others are positive regulators of the IFN response. Therefore, viruses have developed strategies to evade or counteract TRIM proteins, and some even hijack certain TRIM proteins to their advantage. In this review, we summarize the current state of knowledge on the interactions between flaviviruses and TRIM proteins, covering both direct and indirect antiviral mechanisms.

## 1. Introduction

Flaviviruses are viruses belonging to the genus *Orthoflavivirus* (hereafter referred to as flaviviruses for simplicity), within the family *Flaviviridae*. Most of them are arthropod-borne viruses (arboviruses), with the two most common vectors being mosquitoes and ticks [1,2]. Mosquito-borne flaviviruses include a large number of emerging or re-emerging viruses, including dengue virus (DENV), yellow fever virus (YFV), Zika virus (ZIKV), West Nile virus (WNV), and Japanese encephalitis virus (JEV) [2]. The natural cycle of these viruses involves vertebrates as reservoirs and amplifying hosts, and mosquitoes as vectors. Whereas DENV, ZIKV and YFV use nonhuman primates [3], WNV and JEV mostly use wild birds as reservoir hosts [4]. Tick-borne viruses are also drawing increasing attention from health authorities, and comprise several viruses that are pathogenic to humans, including tick-borne encephalitis virus (TBEV) or Kyasanur Forest disease virus (KFDV), which both use rodents as reservoir hosts [5]. Flaviviruses are emerging or re-emerging worldwide, posing a major threat to human health [2,6,7].

Flaviviruses are enveloped viruses with a positive single-stranded RNA genome. They enter into cells by endocytosis followed by pH-triggered fusion with the endosome membrane [8]. Once the genome has been released in the cytoplasm, it is translated at the endoplasmic reticulum (ER) as a polyprotein, then cleaved by viral and cellular proteases. Genome replication and viral assembly take place in invaginations of the ER that constitute ”viral factories”, a cloaking strategy enabling them to replicate away from cellular immune sensors [9]. The viral particle is then matured in the Golgi and released by exocytosis at the plasma membrane [10]. As with all viruses, cellular defenses exist to limit flavivirus replication and, thus, prevent their propagation in the organism. These defenses can be divided into two groups: intrinsic defenses, consisting of restriction factors constitutively expressed in the cell, and the innate immune response, triggered by type I interferon (IFN-I). The IFN-I response is initiated by the recognition of viral RNA by cellular sensors, including RIG-I (retinoic acid-inducible gene I), which triggers a signaling cascade leading to IFN-I production [11]. Once secreted, IFN-I acts both in an autocrine and paracrine manner by binding to its receptor, triggering the so-called JAK/STAT signaling pathway that leads to the expression of hundreds of interferon-stimulated genes (ISGs), the products of which are able to interfere with viral replication [12].

Tripartite motif (TRIM) proteins play a key role in cellular antiviral defenses against viruses, since they include restriction factors, antiviral ISGs, and are also important regulators of the IFN response (reviewed in [13]). TRIM proteins are constitutively expressed at different levels depending on cell type [14], sometimes sufficiently to exert a potent antiviral activity, as in the case for TRIM5α, a potent restriction factor of HIV-1 (human immunodeficiency virus 1) [15]. Moreover, the expression of many TRIM proteins was shown to be induced by IFN-I [14,16,17], but also by IFN-II [14,16], IFN-III, or interleukin 27 [16]. The TRIM protein family comprises proteins with the same tripartite organization, consisting of an RING domain, one or two B-Box domains, and a coiled-coiled (CC) domain [18,19]. In humans, the TRIM protein family comprises over 70 members, all sharing the same tripartite organization but differing from one another in the nature of their C-terminal region. In 2006, Short and Cox proposed a classification of TRIM proteins into nine different subgroups, based on the nature of their C-terminal domains [20]. Interest in this family of proteins surged following the discovery of TRIM5α as a host-dependent restriction factor capable of blocking replication of HIV-1 and other retroviruses [15,21]. Indeed, other TRIM proteins were subsequently shown to interfere with the replication of other viruses, and many of them have been identified as ISGs, both in mouse [17] and human cells [14]. In addition, many TRIM proteins have been identified as positive or negative regulators of the IFN response, via the ubiquitin ligase activity carried by their RING domain [13,22,23].

Over the years, TRIM proteins have, thus, emerged as key players in cellular defenses against many viruses, either by directly interfering with their replication or by modulating the IFN response. In this review, we focus on the antiviral activity of TRIM proteins against members of the *Orthoflavivirus* genus. We summarize the current state of knowledge on the role of the various TRIM proteins in the flavivirus replication cycle, dividing them according to their mechanism of action, in order to distinguish those that interact directly with flavivirus components from those that modulate the IFN response during flavivirus infections.

## 2. TRIM Proteins Directly Inhibit Flaviviruses via Their Ubiquitin-Ligase Activity

**Figure 1:**
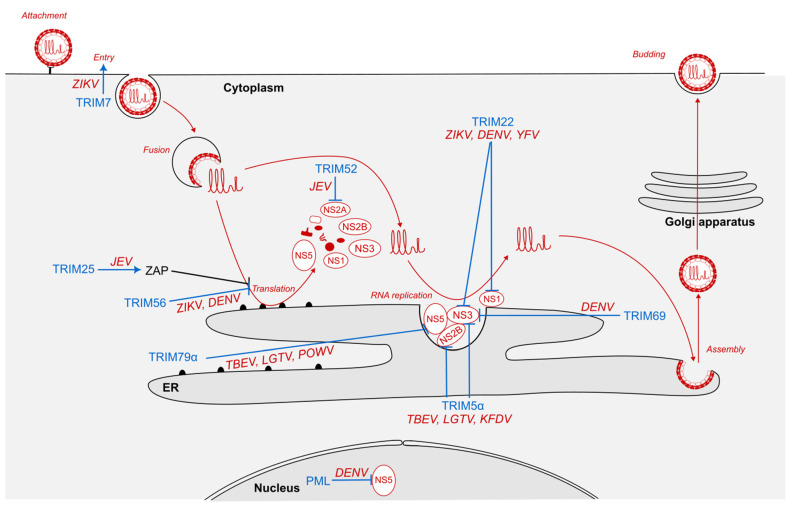
Impact of TRIM proteins on flavivirus replication cycle. Following their interaction with cell receptors, flaviviruses penetrate into target cells by clathrin-dependent endocytosis. The low pH in late endosomes triggers fusion of the viral envelope and endosomal membranes, leading to release of the viral genome into the cytoplasm. At the ER, viral proteins are translated by host ribosomes into polyproteins, which will then be cleaved by cellular and viral proteases. Viral RNA replication and assembly takes place within invaginations of the ER membrane. Immature virions then transit through Golgi apparatus, before mature viruses are released from the cell. ER, endoplasmic reticulum. ZIKV, Zika virus. JEV, Japanese encephalitis virus. DENV, dengue virus. TBEV, tick-borne encephalitis. LGTV, Langat virus. POWV, Powassan virus. KFDV, Kyasanur Forest disease virus.

Ubiquitination is a post-translational modification involving the covalent conjugation of ubiquitin moieties to lysine residues of a specific protein. Protein ubiquitination is a multistep process requiring the sequential action of 3 enzymes: an E1 activating enzyme, an E2 conjugating enzyme, and, finally, an E3 ligase, which confers specificity by transferring ubiquitin to the target protein [24]. Since ubiquitin contains seven lysines, it can itself serve as a substrate for ubiquitination, leading to the formation of polyubiquitin chains. Each type of chain has specific consequences for the substrate protein, with the most frequent being K48- and K63-linked polyubiquitin chains. Whereas K48 polyubiquitination is usually followed by the proteasomal degradation of target proteins, K63 polyubiquitination is generally involved in protein activation [24]. In addition, some TRIM proteins, including TRIM6, can synthesize unanchored K48-linked polyubiquitin chains, which stimulate IFN-mediated antiviral response [25]. Most TRIM proteins display an E3-ubiquitin ligase activity and are, therefore, able to ubiquitinate cellular, but also viral, proteins [13,26]. This activity has been largely described as key for TRIM-mediated restriction of diverse viruses, and flaviviruses seem to be no exception. In particular, certain TRIM proteins have been shown to induce the degradation of certain viral proteins through ubiquitination (Figure 1).

### 2.1. TRIM69

TRIM69 contains a PRY/SPRY domain in its C-terminal part, and belongs to the class IV of TRIM protein family according to the Short and Cox classification [20]. Like most proteins in its family, TRIM69 displays a well-characterized E3-ubiquitin ligase activity supported by its RING domain, and localizes both in the cytoplasm and the nucleus. Interestingly, its nuclear localization has been shown to be dependent on its RING domain [27]. Although it was first discovered in testis [27], TRIM69 is expressed in a variety of cell types, including primary blood cells [28]. TRIM69 has been described as a broad-spectrum antiviral protein, capable of inhibiting unrelated viruses, including vesicular stomatitis virus (VSV) in human cell lines [29], HIV-1, and severe acute respiratory syndrome coronavirus 2 (SARS-CoV-2) in macrophage-like cells [28]. In the case of HIV-1 and SARS-CoV-2, restriction has been shown to involve the association of TRIM69 with microtubules, inducing their stabilization [28].

TRIM69 also inhibits flavivirus replication by targeting their NS3 protein (Figure 1). NS3 is a nonstructural protein of flaviviruses with both protease and helicase activities [30,31]. As such, NS3 is particularly crucial for the cleavage of the polyprotein and the replication of the viral RNA. TRIM69 expression is induced by IFN-I treatment [14] but has also been shown to be upregulated upon DENV (serotype 2) infection in different human and mouse cell lines, in which it inhibits DENV replication [32]. In vivo, mice lacking TRIM69 displayed a lower resistance to DENV infection [32]. This restriction was shown to rely on its E3-ubiquitin ligase activity and, therefore, on its RING domain. Mechanistically, TRIM69 was shown to interact with DENV NS3 protein, inducing its ubiquitination on a specific residue (Lys104), leading to NS3 proteasomal degradation both in mice and human cells [32]. Of note, a more recent study mapped the interface of interaction between TRIM69 and DENV NS2B/NS3 complex [33]. It proposed a rationale for the ubiquitination of NS3 Lys104 by TRIM69 and underlined that both Lys104 and residues at the interface were conserved across DENV serotypes but also in ZIKV, JEV, and WNV, suggesting that TRIM69 could be involved in restricting other flaviviruses [33].

### 2.2. TRIM22

TRIM22 is also a member of the class IV subfamily of TRIM proteins (with a PRY/SPRY domain in its C-terminal part), which displays an E3-ubiquitin ligase activity. Its localization is predominantly nuclear, although diffuse cytoplasmic expression has also been observed in certain cell types [34]. TRIM22 is ubiquitously expressed [35,36] and has been shown to restrict viruses from different families (reviewed in [37]). This inhibition involves different mechanisms depending on the virus considered. For example, its nuclear localization has been shown to be important for its antiviral activity against Herpes Simplex Virus 1 and Porcine Reproductive and Respiratory Syndrome Virus (PRRSV) [38,39], while its E3-ubiquitin ligase activity is required for viral protein degradation by the ubiquitin-dependent proteasome in the case of SARS-CoV-2 [40], Influenza A virus (IAV) [41], or Hepatitis C Virus (HCV, a member of the *Hepacivirus* genus within the *Flaviviridae* family) [42].

In the case of flaviviruses, TRIM22 has been shown to inhibit DENV, YFV, and ZIKV replication [43]. By interacting with both NS1 and NS3 via its SPRY domain (Figure 1), it induces their ubiquitination by K48-linked chains, thus triggering their degradation by the proteasome [43].

### 2.3. TRIM5α

TRIM5α has been extensively studied for its ability to block HIV-1 replication, which it does by inducing capsid disassembly, thus inhibiting reverse-transcription and subsequent steps of replication [15]. Whether or not the ubiquitin ligase activity carried by TRIM5α’s RING domain is involved in HIV-1 restriction remains unclear [44,45]. TRIM5 has also been shown to restrict Vaccinia virus [46] and Epstein–Barr virus (EBV) [47], each time relying on its RING domain. TRIM5α belongs to the class IV TRIM proteins, is ubiquitous, and mainly localizes in the cytoplasm of cells, were it forms highly dynamic speckles [18].

Among flaviviruses, human and rhesus monkey TRIM5α restrict the replication of tick-borne encephalitis virus (TBEV), Langat virus (LGTV), and Kyasanur Forest disease virus (KFDV) [48]. TRIM5α was found to reduce TBEV, LGTV, and KFDV replication in human cell lines and primary dendritic cells, both in overexpression and knockdown models. It was shown to interact with the viral NS2B/NS3 complex (Figure 1), leading to its ubiquitination by K48-linked chains that target the viral proteins to the proteasome for degradation. Surprisingly, TRIM5α could not restrict any mosquito-borne flaviviruses tested (DENV, ZIKV, YFV, and WNV) [48], and may, thus, constitute a specific inhibitor of tick-borne flaviviruses.

### 2.4. TRIM52

TRIM52 belongs to the class V subfamily of TRIM proteins, as it lacks a specific C-terminal domain. TRIM52 displays an E3-ubiquitin ligase activity, and is ubiquitously expressed both in the cytoplasm and the nucleus [49,50]. TRIM52 has not been previously described as an antiviral factor, but was shown to activate the NF-κβ signaling pathway, thus triggering inflammatory response [50].

The same team demonstrated that TRIM52 efficiently inhibited JEV replication when overexpressed in different human cell lines [49]. It was found to target JEV NS2A (Figure 1), a small nonstructural viral protein involved in viral replication, assembly, and inhibition of innate immunity (reviewed in [51]). The proposed mechanism is a TRIM52-mediated ubiquitination of JEV NS2A with K48-linked chains, leading to its proteasomal degradation [49].

### 2.5. TRIM7

TRIM7 is a cytoplasmic protein [52,53] expressed in most human tissues [54]. A member of the class IV of TRIM proteins, TRIM7 displays a well-characterized E3-ubiquitin ligase activity, which allows it to play a role in multiple innate immune pathways (reviewed in [55]), thus restricting several unrelated viruses, including members of the *Picornaviridae* and *Caliciviridae* viral families [52,56,57].

Concerning flaviviruses, the only study to date concerns ZIKV and, surprisingly, describes TRIM7 as proviral [53]. Specifically, this study from Giraldo and colleagues demonstrated that ZIKV could take advantage of TRIM7 ubiquitin-ligase activity [53]. They showed that the ZIKV E protein could be ubiquitinated by TRIM7, especially on its K38 residue. This ubiquitination with K63-linked chains resulted in a higher infectivity of ZIKV virions through more efficient attachment and entry steps into host cells (Figure 1) [53]. Importantly, these observations have been confirmed in vivo, since in mice, ubiquitination of ZIKV E protein by TRIM7 results in greater viral replication, especially in the brain and reproductive organs [53]. These data suggest that TRIM7 might play a role in ZIKV tropism in mammals, as it is well known that ZIKV is a neurotropic virus transmitted through the placenta from mother to infant and causes children microcephaly [58]. Strikingly, ZIKV E ubiquitination showed no impact on viral replication in mosquitoes (both in vitro and in vivo) and was only partial in virions produced from mosquito cells, emphasizing the importance of host-specific modification of virions in viral cycle and infectivity [53].

## 3. TRIM Proteins Directly Inhibit Flavivirus Replication via a Ubiquitin-Independent Mechanism

Although targeting viral proteins to the proteasome is an efficient way to restrict viral replication, it is not the only mechanism through which TRIM proteins interfere with flavivirus infection.

### 3.1. PML (TRIM19)

PML (promyelocytic leukemia protein, also termed TRIM19) has no specific C-terminal domain, which classifies it as a TRIM protein of subgroup V. It is ubiquitously expressed as seven different isoforms in human cells, which are designated PMLI to VII. With the notable exception of PML VIIb, PML isoforms localize in the nucleus, where they are the major component of PML nuclear bodies (PML NBs) [59]. PML-NBs have been widely characterized, notably for their role in innate immune response [60] and viral infections [61].

In a first article, Giovannoni and colleagues demonstrated that PML restricts DENV (serotype 2) infection in A549 cells [62]. A disruption of PML-NBs was also observed during the course of DENV infection, although it was not investigated whether this was due to the infection itself. Finally, they showed that IFN-I treatment reduced DENV infection while increasing the number of PML-NBs in infected cells. In a follow-up study, the same group proved that PML interacts with DENV NS5 and recruits it into PML-NBs (Figure 1) [63]. This sequestration of DENV polymerase in PML-NBs impaired DENV replication but could only be achieved by isoforms III and IV of PML. In parallel, overexpression of DENV NS5 reduced the number of PML-NBs and increased the turnover of isoforms III and IV of PML, suggesting a viral mechanism for counteracting this cellular defense. Remarkably, a similar mechanism was recently described with porcine PML isoforms I, III, IV, and V, which are able to inhibit JEV replication in a porcine cell line [64].

### 3.2. TRIM56

A member of the class V subfamily of TRIM proteins, TRIM56 is known to localize in the cytoplasm of cells of most human tissues [65,66] and to exhibit an E3 ubiquitin-ligase activity [65]. This activity is notably required for TRIM56 antiviral activity against the bovine viral diarrhea virus (BVDV), a pestivirus member of the *Flaviviridae* family [65]. Moreover, TRIM56 has been characterized as a positive regulator of the Toll-like receptor 3 (TLR3) signaling pathway, responsible for dsRNA sensing and further activation of the antiviral response [67], a feature that enables it to inhibit porcine epidemic diarrhea virus (PEDV), a member of the coronavirus family [68]. It has also been described to restrict hepatitis B virus (HBV), through the activation of the NF-κβ signaling pathway [69] and to enhance the activation of cGAS, a cytoplasmic sensor of DNA [70].

TRIM56 has been shown to inhibit YFV and DENV (serotype 2) replication in different human cell lines when overexpressed, and to promote it when silenced [71]. Kinetics led the authors to conclude that the restriction occurred at the step of viral RNA replication [71]. Moreover, this restriction was shown to be strictly dependent on both its RING and C-terminal domain. Finally, by silencing effectors of the innate immune response, the authors demonstrated that TRIM56 inhibition of YFV and DENV did not require RIG-I, TRIF, or STING, thus suggesting that TRIM56 would act directly on the virus to block its replication, although no mechanism was proposed (Figure 1) [71]. TRIM56 was also found to inhibit ZIKV replication, using both overexpression and knockdown models, in HeLa but also in neural cell lines [72]. This antiviral activity relied on a direct interaction between the C-terminal domain of TRIM56 and ZIKV RNA, a mechanism reminiscent of TRIM56-mediated inhibition of IAV [73]. Furthermore, ZIKV inhibition (but not the binding) was shown to be dependent on TRIM56 E3-ligase activity mediated by its RING domain. Knowing that TRIM56 shows sequence homology with the NHL domain of other TRIM proteins, a domain involved in miRNA regulation [74], they hypothesized that TRIM56-mediated restriction of ZIKV might be linked to miRNA processes, but proved that it was not the case, although the precise mechanism was not deciphered. TRIM56 showed a similar inhibition of DENV (serotype 1) [72].

### 3.3. TRIM25

TRIM25 belongs to the class IV subfamily of TRIM proteins and possesses a well-described E3-ubiquitin ligase activity [75]. TRIM25 is a ubiquitously expressed cytoplasmic protein [18]. It was found to inhibit rabies virus [76] and influenza virus [77] by directly targeting viral components. TRIM25 has been shown to be a crucial cofactor for ZAP (Zinc-Finger Antiviral Protein), notably for the inhibition of Sindbis [78] or Ebola [79] viruses.

This partnership between TRIM25 and ZAP is also essential for TRIM25-mediated restriction of JEV RNA translation (Figure 1) [80]. In this context, ZAP (but not TRIM25) bound viral RNA to inhibit its translation, and was potentiated by the interaction with TRIM25, although no mechanism was proposed (Figure 1). TRIM25 was also highlighted as an antiviral against WNV both in gain-of-function [81] and loss-of-function [82] screens, as well as against YFV [81] and ZIKV [83] in two gain-of-function screens.

However, it is important to note that TRIM25 is also an important regulator of the innate immune response and is, thus, capable of exerting broad-spectrum antiviral activity via an enhancement of the IFN response. This aspect will be discussed in Section 4.2.

### 3.4. TRIM79α

TRIM79α (also called TRIM30-3 or TRIM30D) is a mouse-specific ISG described in 2011, which belongs to the class IV subfamily of TRIMs [84]. It localizes in the cytoplasm of cells [84] and is expressed in most mouse tissues, although it is higher in immune organs like the spleen or bone marrow [84].

Taylor and colleagues established that TRIM79α interacts with LGTV NS5 (Figure 1), the viral polymerase, promoting its degradation by lysosomes, cell compartments that degrade large protein complexes [84]. Interestingly, NS3 and NS2B were also degraded by TRIM79α as part of the replication complex. This resulted in the impairment of LGTV replication when TRIM79α was overexpressed, or in its enhancement in a knockdown model [84]. Contrary to proteasome-dependent degradations described above, ubiquitination is not required for TRIM79α to promote the lysosomal degradation of NS5. TBEV and Powassan virus (POWV), another tick-borne flavivirus, were also shown to be inhibited by the same mechanism, as opposed to mosquito-borne WNV and JEV, that are resistant to TRIM79α-mediated restriction [84].

TRIM proteins are, therefore, able to block flaviviruses replication at different steps of their cycle (Figure 1). Yet, some flaviviruses have evolved mechanisms to counteract TRIM protein-mediated restriction, or even to take advantage of TRIM protein activities to enhance replication. Nevertheless, TRIM proteins do not always act directly on viral components, but are also part of a broader antiviral response against flaviviruses.

## 4. TRIM Proteins Restrict Flaviviruses Replication via IFN Response Modulation

**Figure 2:**
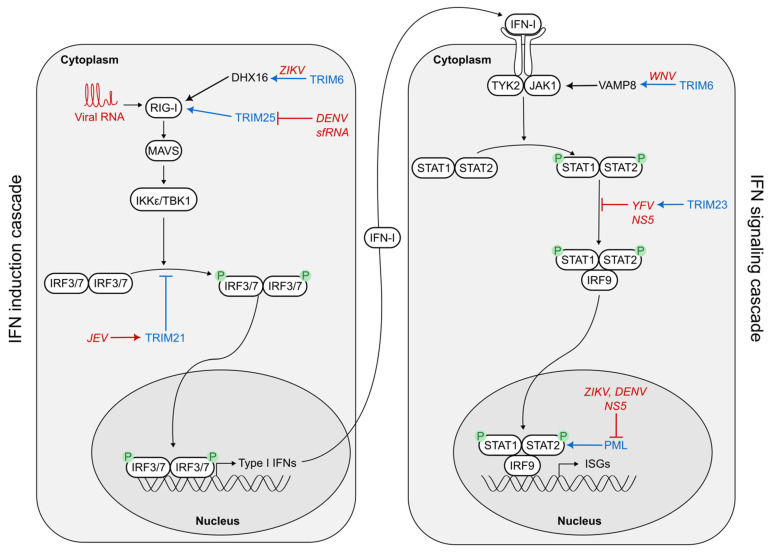
Modulation of type I interferon response by TRIM proteins upon flavivirus infection. Upon its activation by viral RNA, RIG-I interacts with the downstream adaptor MAVS, which in turn recruits TANK-binding kinase 1 (TBK1) and IκB kinase-ε (IKKε), leading to the phosphorylation of interferon Regulatory Factor 3 and/or 7 (IRF3/7). Once phosphorylated, IRF3/7 dimerize and translocate into the nucleus to induce the expression of IFN-I. Once secreted, IFN-I binds to its receptor, leading to the activation of the Jak tyrosine kinases Tyk2 and JAK1, which in turn phosphorylate STAT1 and STAT2. Phosphorylated STATs heterodimerize and associate with IFN regulatory factor 9 (IRF9), to form a complex known as IFN-stimulated growth factor 3 (ISGF3). Once this complex translocates into the nucleus, it induces the expression of hundreds of interferon-stimulated genes (ISGs). Cellular and viral proteins that interfere with the type I IFN response are indicated in blue and red, respectively. IFN, interferon; ZIKV, Zika virus; JEV, Japanese encephalitis virus; DENV, dengue virus; WNV, West Nile virus.

One of the first lines of antiviral defenses is the IFN-I response. This response is triggered by the recognition of pathogen-associated molecular patterns (PAMPs) by pathogen recognition receptors (PRRs), including the cytoplasmic RNA sensors RIG-I and MDA5 (Figure 2). This recognition initiates a signaling cascade (the IFN induction cascade), which results in the phosphorylation and subsequent translocation of IRF3 and/or IRF7 into the nucleus to trigger the expression of IFN-I. IFN-I is then secreted, binds to its receptor IFNAR, and activates the JAK-STAT signaling cascade (the so-called IFN signaling cascade). STAT1/2 are phosphorylated and form a complex with IRF9, the so-called ISGF3 complex. This complex is translocated to the nucleus, leading to the expression of hundreds of ISGs that confer an antiviral state to the cells (Figure 2) [12].

The IFN-I response is essential for the organism to control viral infections and is, therefore, tightly regulated. In this context, TRIM proteins have recently emergent as important regulators of these antiviral pathways [13,85]. Given the key role of TRIM proteins in the IFN response, it is no surprise that they represent a prime target for viruses, and flaviviruses in particular, to counteract cellular defenses (Figure 2).

### 4.1. TRIM6

TRIM6 is ubiquitously expressed and forms speckles in the cytoplasm of cells [18]. It belongs to class IV of TRIM proteins and exhibits E3-ubiquitin ligase activity [25]. It is through this activity that TRIM6 promotes Ebola virus replication, by regulating its transcription and assembly [86,87]. It has also been established that TRIM6 can stimulate the IFN signaling cascade via the synthesis of unanchored ubiquitin chains that interact with IKKε, a kinase that phosphorylate STAT1, and which is required for IKKε-dependent ISG expression [25]. Interestingly, this feature is counteracted by Nipah virus [88].

To date, TRIM6 is the only TRIM protein described as being capable of controlling flavivirus replication by acting on both IFN induction and signaling cascades (Figure 2). In the context of ZIKV infection, it has been reported that the RNA helicase DHX16 is able to bind viral RNA and to form a complex with RIG-I to ensure an optimal production of IFN-I [89]. The formation of this complex is enhanced by an interaction between DHX16, the SPRY domain of TRIM6, and unanchored K48-linked polyubiquitin chains synthesized by TRIM6 (Figure 2) [89]. Therefore, the E3-ubiquitin ligase activity of TRIM6 is essential for an efficient antiviral IFN-I response against ZIKV. In the context of WNV infection, TRIM6 was also demonstrated to enhance ISG response by targeting the IFN signaling cascade [90]. In this study, VAMP8 (Vesicle Associated Membrane Protein 8) was found to be downregulated upon TRIM6 knockdown. Further experiments showed that VAMP8 was important for JAK1 phosphorylation, and, thus, activation of the IFN signaling cascade (Figure 2). Moreover, both VAMP8 expression and VAMP8-mediated phosphorylation of JAK1 were enhanced by TRIM6 [90]. It was also shown that VAMP8 and TRIM6 interact, but the exact mechanism remains to be elucidated [90].

### 4.2. TRIM25

Beyond its capacity to directly inhibit replication of several viruses, as discussed above (Section 3.3), TRIM25 is also an important positive regulator of RIG-I signaling. Indeed, TRIM25, upon its own deubiquitylation, polyubiquitinates RIG-I, thus promoting its sustained activation, leading to greater IFN-I production and, thus, enhancing the antiviral response [75]. For instance, this mechanism has proved efficient to block Sendai virus [75], porcine reproductive and respiratory syndrome virus (PRRSV) [91], or thrombocytopenia syndrome virus (SFTSV) [92], although most viruses have evolved mechanisms to counteract TRIM25-mediated enhancement of RIG-I activation.

A similar conclusion has been reached concerning DENV (serotype 2) restriction by TRIM25 [93]. Indeed, comparing two strains isolated in Puerto Rico, Manokaran and colleagues showed that higher production of subgenomic flaviviral RNA (sfRNA), which are small RNAs produced by incomplete digestion of flaviviral genomic RNA by the cellular exoribonuclease XRN1, correlated with a lower IFN-I response during infection [93]. They then established that DENV sfRNA binds TRIM25 and interferes with its deubiquitylation, thus decreasing RIG-I activation and IFN-I induction efficacy (Figure 2) [93]. Interestingly, these results show that DENV, but also other viruses from distant viral families, has evolved mechanisms to silence IFN-I response at its very first step, allowing stronger replication.

### 4.3. TRIM21

TRIM21, another member of class IV of TRIM proteins, is ubiquitously expressed in the cytoplasm of cells and displays an E3-ubiquitin ligase activity [18]. It has been identified as an antiviral protein against viruses from various families, notably via a ubiquitin-dependent degradation of viral proteins of HBV [94,95] or IAV [96], for example. TRIM21 has also been shown to be involved in the regulation of the IFN-I signaling pathway by targeting IRF7 [97] and IRF3 [98]. Concerning IRF3, a first study demonstrated that TRIM21 interacts with IRF3 via its C-terminal SPRY domain and induces its ubiquitin-dependent proteasomal degradation upon LPS, poly I:C, and Sendai virus stimulations, thus inhibiting IFN-I antiviral response [98]. However, a year later, a contradictory paper described that TRIM21 sustains the activation of IRF3 upon infection by Sendai virus by inhibiting its ubiquitination, thus preventing its degradation [99].

In the case of flaviviruses, TRIM21 has been identified as an antiviral against WNV in a loss-of-function screen by shRNA in HeLa cells [82]. Conversely, TRIM21 has been demonstrated to be proviral in the context of JEV infection of microglial cells [100]. Indeed, in human microglial cells (but not in HeLa cells), JEV infection strongly enhanced TRIM21 expression, leading to a reduction in IRF3 phosphorylation, therefore impairing the production of IFN-I in infected cells (Figure 2) [100]. The precise mechanism was not dissected further, but the study clearly highlights potential proviral effects of TRIM proteins modulation of IFN-I response [100].

### 4.4. TRIM23

TRIM23 contains an ARF domain in its C-terminal part, which confers GTPase activity [101] in addition to its E3 ubiquitin-ligase activity [102], making it the only member of the class IX subgroup of TRIM proteins [20]. TRIM23 is a ubiquitous protein with both nuclear and cytoplasmic localization [18]. It has been described as an important modulator of various pathways upon viral infections, including the NF-κβ pathway in the case of human cytomegalovirus infection [103] or virus-induced autophagy [104]. TRIM23 has also been shown to enhance NF-κβ signaling by ubiquitination of NEMO, an adaptor of this signaling pathway [105].

But the role of TRIM23 upon flavivirus infection involves another innate immune pathway: the IFN-I signaling cascade. In particular, YFV NS5 has been found to be ubiquitinated on its K6 residue by TRIM23 with K63-linked ubiquitin chains [102]. This ubiquitination enabled YFV NS5 to bind STAT2 (when it is associated to STAT1), leading to the formation of an NS5-STAT1/2 complex (Figure 2). This interaction impairs the capacity of STAT1/2 to form a complex with IRF9, to translocate in the nucleus and to trigger the expression of ISGs [102]. Therefore, TRIM23 has a proviral effect upon YFV infection, as NS5 takes advantage of TRIM23’s enzymatic activity to shut down IFN response (Figure 2).

### 4.5. PML (TRIM19)

As explained above (see Section 3.1), PML is the major component of PML-NBs and was shown to inhibit DENV through a direct interaction with its NS5 protein [63]. The interaction between PML and NS5 was also shown to be involved in the inhibition of IFN signaling cascade in hBMECs (human brain microvascular endothelial cells) upon DENV or ZIKV infection [106]. NS5 localizes to the nucleus and interacts with SUMO proteins [106,107]. This interaction was shown to disrupt the interaction of SUMO with PML and STAT2, its usual partners in NBs, leading to decreased ISG production (Figure 2). Although the mechanism was not precisely described, this study highlights an original mechanism developed by DENV and ZIKV to attenuate the IFN-mediated antiviral response [106].

TRIM proteins, thus, modulate the IFN response in the context of flavivirus infection, although not always to the advantage of the host cell (Figure 2). Indeed, while some TRIM proteins potentiate IFN induction and/or signaling cascades, others can be hijacked by flaviviruses to dampen the IFN response, thus illustrating the evolutionary arms race between TRIM proteins and flaviviruses.

## 5. Conclusions and Perspectives

Given the threat they pose to public health worldwide, emerging and re-emerging flaviviruses are the focus of ever-increasing attention [2]. Especially, their interactions with human intrinsic and innate defenses have gained interest in the last few decades, as illustrated by the growing interest in TRIM proteins in the context of flaviviral infections (Table 1). While TRIM proteins are largely constitutively expressed and can, therefore, be involved in so-called intrinsic antiviral immunity, most of them are ISGs, whose expression can be induced by different types of IFN [14,16,17]. Unlike other ISGs, TRIM proteins have the unique ability not only to directly block replication of various viruses, but also to stimulate the innate immune response, making them powerful broad-spectrum antivirals. A perfect illustration of the impact of TRIM proteins on virus propagation is the fact that most viruses have had to develop escape or counteract mechanisms in order to replicate. Some viruses have even managed to hijack the functions of TRIM proteins to their advantage. This suggests that flaviviruses and human TRIM proteins have coevolved and are probably still engaged in an arms race [108]. Understanding these complex interactions is not only a challenge for fundamental virology, but could also lead to the identification of vulnerabilities in flavivirus replication, which could be exploited for therapeutic strategies.

Although the picture of interactions between flaviviruses and TRIM proteins is far from complete, there is a clear overall difference in TRIM restriction between mosquito-borne and tick-borne flaviviruses, although the latter are generally less studied. In particular, TRIM79α [84] and TRIM5α [48] were shown to restrict the replication of most tick-borne viruses, but none of the mosquito-borne flaviviruses tested (Table 1). Given their relative phylogenetic distance among flaviviruses [1], this difference is not surprising. Moreover, other differences in host–pathogen interactions between tick-borne and mosquito-borne flaviviruses have been described, as illustrated by the recent description of the unique mechanism by which the NS5 protein encoded by tick-borne flaviviruses inhibits IFN signaling via an interaction with TYK2 [109]. These differences could represent an adaptation of the viruses to their respective vectors, and raise many questions about the importance of the vector in viral infectivity in humans. In this respect, it has been shown that the ubiquitination of the ZIKV E protein differs depending on whether the virus originates from mammalian or mosquito cells, affecting its overall pathogenesis [53]. Similarly, it has been described that the glycosylation of flavivirus envelope differs between human and mosquito cells, impacting their infectivity [110]. It would also be of great interest to assess whether TRIM proteins of different hosts, especially flavivirus amplifying host, can inhibit their replication and to what extent this would be conserved in humans. Two articles have recently showed that some porcine PML isoforms play a role in inhibiting JEV [64,111] and another one has focused on duck TRIM25, which was shown to restrict the Tembusu virus (an arbovirus from the *Orthoflavivirus* genus). These pave the way for future research into TRIM-mediated immunity in birds and nonhuman mammals, as a way to better understand overall flavivirus pathogenesis.

## Figures and Tables

**Table 1:** Summary of the pro- and antiviral effects of TRIM proteins on flaviviruses. DENV, dengue virus. ZIKV, Zika virus. YFV, yellow fever virus. JEV, Japanese encephalitis virus. WNV, West Nile virus. TBEV, tick-borne encephalitis. LGTV, Langat virus. KFDV, Kyasanur Forest disease virus. POWV, Powassan virus. n/a, nor applicable. IFN-I, type I interferon.

Arthropod Vector	Targeted Flavivirus	TRIM Protein	Pro- (+) or Anti- (−) Viral	Mechanism	Ubiquitin-Dependent	References
Mosquito	DENV	TRIM69	−	Direct (NS3 degradation)	yes	[32,33]
		TRIM22	−	Direct (NS1 and NS3 degradation)	yes	[43]
		TRIM19	−	Direct (NS5 sequestration)	no	[63]
		TRIM56	−	Not described	n/a	[71]
		TRIM25	−	Indirect (pro-IFN-I)	yes	[93]
		TRIM19	−	Indirect (pro-IFN-I)	no	[106]
	ZIKV	TRIM22	−	Direct (NS1 and NS3 degradation)	yes	[43]
		TRIM7	+	Direct (Env ubiquitination)	yes	[53]
		TRIM56	−	Not described	n/a	[72]
		TRIM6	−	Indirect (pro-IFN-I)	yes	[89]
		TRIM19	−	Indirect (pro-IFN-I)	no	[106]
	YFV	TRIM22	−	Direct (NS1 and NS3 degradation)	yes	[43]
		TRIM56	−	Not described	n/a	[71]
		TRIM23	+	Indirect (anti-IFN-I)	yes	[102]
	JEV	TRIM52	−	Direct (NS2A degradation)	yes	[49]
		TRIM25	−	Not described (involves ZAP)	no	[80]
		TRIM21	+	Indirect (anti-IFN-I)	n/a	[100]
	WNV	TRIM6	−	Indirect (pro-IFN-I)	n/a	[90]
Tick	TBEV	TRIM5α	−	Direct (NS3 degradation)	yes	[48]
		TRIM79α	−	Direct (NS5 degradation)	no	[84]
	LGTV	TRIM5α	−	Direct (NS3 degradation)	yes	[48]
		TRIM79α	−	Direct (NS5 degradation)	no	[84]
	KFDV	TRIM5α	−	Direct (NS3 degradation)	yes	[48]
	POWV	TRIM79α	−	Direct (NS5 degradation)	no	[84]
